# Dunglison’s Medical Dictionary

**Published:** 1852-05

**Authors:** 


### DUNGLISON'S MEDICAL DICTIONARY.

We have received from the publishers a copy of this valuable dictionary, which has reached the eighth edition. It was a valuable work at the start, and each reprint has been improved as well as enlarged. We assure any reader who may not have examined it that he could not possess himself of a more useful medical lexicon.

